# The effect of acute exercise on objectively measured sleep and cognition in older adults

**DOI:** 10.3389/fpsyg.2023.1207199

**Published:** 2023-10-05

**Authors:** Kelsey R. Sewell, Nathan D. W. Smith, Stephanie R. Rainey-Smith, Jeremiah Peiffer, Hamid R. Sohrabi, Kirk I. Erickson, Belinda M. Brown

**Affiliations:** ^1^Centre for Healthy Ageing, Health Futures Institute, Murdoch University, Murdoch, WA, Australia; ^2^School of Medical and Health Sciences, Edith Cowan University, Joondalup, WA, Australia; ^3^Australian Alzheimer’s Research Foundation, Sarich Neuroscience Research Institute, Nedlands, WA, Australia; ^4^School of Psychological Science, University of Western Australia, Perth, WA, Australia; ^5^Department of Biomedical Sciences, Macquarie University, Sydney, NSW, Australia; ^6^Department of Psychology, University of Pittsburgh, Pittsburgh, PA, United States; ^7^PROFITH “PROmoting FITness and Health Through Physical Activity” Research Group, Department of Physical and Sports Education, Faculty of Sport Sciences, Sport and Health University Research Institute (iMUDS), University of Granada, Granada, Spain; ^8^AdventHealth Research Institute, Orlando, FL, United States

**Keywords:** exercise, cognition, sleep, older adults, high intensity

## Abstract

**Background:**

Exercise can improve cognition in aging, however it is unclear *how* exercise influences cognition, and sleep may partially explain this association. The current study aimed to investigate whether objectively measured sleep mediates the effect of an acute exercise intervention on cognition in older adults.

**Methods:**

Participants were 30 cognitively unimpaired, physically active older adults (69.2 ± 4.3 years) with poor sleep (determined via self-report). After a triple baseline cognitive assessment to account for any natural fluctuation in cognitive performance, participants completed either a single bout of 20-minutes of high intensity exercise on a cycle ergometer, or a control condition, in a cross-over trial design. Cognition was measured immediately post-intervention and the following day, and sleep (total sleep time, sleep onset latency, sleep efficiency, % of rapid eye movement sleep, light sleep and deep sleep) was characterized using WatchPAT^™^ at baseline (5 nights) and measured for one night after both exercise and control conditions.

**Results:**

Results showed no effect of the exercise intervention on cognition immediately post-intervention, nor an effect of acute exercise on any sleep variable. There was no mediating effect of sleep on associations between exercise and cognition. However, a change from baseline to post-intervention in light sleep and deep sleep did predict change in episodic memory at the ~24 h post-intervention cognitive assessment, regardless of intervention condition.

**Discussion:**

There was no effect of acute high intensity exercise on sleep or cognition in the current study. However, results suggest that associations between sleep and cognition may exist independently of exercise in our sample. Further research is required, and such studies may aid in informing the most effective lifestyle interventions for cognitive health.

## Introduction

There is a well-established link between habitual physical activity (bodily movement resulting in energy expenditure; Caspersen et al., 1985) and preservation of cognition in aging (Sofi et al., 2011; Blondell et al., 2014; Yoneda et al., 2021; Sewell et al., 2023a). However, evidence for the efficacy of exercise (i.e., structured/planned physical activity) interventions to improve cognitive function in older adults is inconsistent (Sink et al., 2015; Young et al., 2015). Lifestyle and genetic factors may affect individual cognitive response to exercise, contributing to inconsistencies in the literature (Erickson et al., 2022). It is increasingly important to understand factors that influence the exercise-cognition association in order to promote a precision approach to maintaining cognitive health in aging.

Sleep is one factor which may contribute to the strength of cognitive response to exercise interventions (Sewell et al., 2021). Poor sleep is associated with a greater risk of cognitive decline and dementia (Bubu et al., 2016), and increased slow wave activity during sleep may improve memory performance in older adults (Westerberg et al., 2015). Sleep stages may differentially influence aspects of cognitive function, for example rapid eye movement (REM) sleep is important for memory consolidation (Boyce et al., 2017). Exercise interventions can improve sleep outcomes, although this may be subject to specific sleep parameters (Kredlow et al., 2015). For example, acute exercise has been shown to benefit sleep duration, sleep onset latency (time taken to fall sleep), sleep efficiency (% of time in bed spent asleep), stage 1 sleep (light sleep) and slow wave sleep, specifically (Kredlow et al., 2015). Additional research is required to investigate these complex and specific associations between sleep characteristics, exercise and cognitive domains, in order to best preserve cognition in aging.

Few studies to date have investigated associations between sleep, exercise and cognition. However, some observational cross-sectional research indicates that physical activity may compensate for sleep-related cognitive deficits (Lambiase et al., 2014; Nakakubo et al., 2017; Sewell et al., 2023b). To the authors’ knowledge, only one experimental study has examined the effect of an acute exercise intervention on sleep and cognition in cognitively unimpaired older adults (Won et al., 2019). Won et al. found that longer habitual sleep duration was associated with better executive function post-exercise, however, there was no relationship between exercise and executive function when sleep was not considered (Won et al., 2019). Based on these results, sleep duration may not only impact the strength of association between exercise and cognition, but also, sleep may partially explain this association; a paradigm which has been further investigated in observational studies.

Results from cross-sectional studies indicate self-reported and objectively measured sleep may partially explain the association between physical activity and cognition (Wilckens et al., 2018; Cheval et al., 2022). However, another observational study showed no mediating effect of self-reported sleep quality on the association between self-reported physical activity and global cognition (Yuan et al., 2020). Many variables may explain these disparate findings, specifically, self-reported vs. objectively measured sleep and physical activity, and differences in the parameter of focus for each variable (i.e., sleep efficiency vs. overall quality, physical activity frequency vs. total volume). Currently, there are no published experimental studies which examine the potential mediating effect of sleep on the exercise-cognition association, specifically measuring sleep staging (e.g., deep sleep, light sleep, REM sleep) and assessing multiple cognitive domains (e.g., attention, memory). Additionally, no study has examined these relationships with consideration of exercise intensity, which may influence exercise-induced sleep and cognitive change, specifically such that high-intensity exercise may produce the greatest benefit (Angevaren et al., 2007; Brown et al., 2012; Larsen et al., 2019; Oberste et al., 2019). Thus, additional exercise intervention studies utilizing objective sleep measures, and considering exercise intensity, are required to corroborate cross-sectional observational findings of a mediating role for sleep in the exercise-cognition relationship.

The current study was intended as a proof-of-concept trial to examine the effect of a high intensity acute exercise intervention on objectively measured sleep variables (total sleep time; TST, sleep efficiency, time spent in light sleep, deep sleep, and REM sleep), and cognition (executive function, memory and attention), in cognitively unimpaired older adults. In prior studies, exercise has been shown to acutely improve both sleep and cognition (Chang et al., 2014; Kredlow et al., 2015), thus an acute intervention was selected due to the novel, proof-of-concept nature of our study. Aim one was to determine whether our high intensity acute exercise intervention improved cognition immediately post-exercise, and aim two was to examine whether our high intensity acute exercise intervention improved sleep after exercise, compared to baseline. We hypothesized that both sleep and cognition would improve post-exercise. To corroborate observational studies discussed above, our third aim (the proof-of-concept aspect) was to determine whether any sleep variable mediated the association between acute high intensity exercise and performance on measures of memory, executive function or attention, the day following exercise. Based on Wilckens et al. (2018), we hypothesized that sleep efficiency would mediate associations between exercise and executive function. Additionally, based on associations with exercise (Kredlow et al., 2015) and memory (Westerberg et al., 2015), we hypothesized that time spent in deep sleep would mediate associations between exercise and memory.

## Method

### Participants

Participants for this study were 30 community-dwelling older adults aged 60–80 years who were physically “active,” cognitively unimpaired, and self-reported poor sleepers. Participants were included if they reported meeting physical activity guidelines of ≥150 min of moderate intensity exercise per week within the last month (Haskell et al., 2007), but not regularly engaging in high intensity exercise. We recruited physically active participants to limit any risks associated with delivering a high intensity exercise intervention to sedentary individuals. Cognitive status was determined by score on the teleMoCA (telephone Montreal Cognitive Assesment, participants must score ≥17; Pendlebury et al., 2013), which was confirmed using the full version of the MoCA in the second cognitive session [inclusion based on Rossetti et al. (2011) norms]. Poor sleep was defined by scoring >5 on the Pittsburgh Sleep Quality Index (PSQI; Backhaus et al., 2002). Eligibility was determined using a semi-structured telephone interview which included collection of medical and demographic information pertaining to exclusion criteria, physical activity level assessment, completion of the teleMoCA and completion of the PSQI electronically. Exclusion criteria included: inability to undertake cycling-based exercise; inability to obtain medical clearance to participate in exercise; current or past history of major psychiatric conditions (e.g., schizophrenia, schizoaffective disorder, or bipolar disorder); untreated sleep apnoea (determined via self-report); shift work; recent travel through more than one time zone; excessive regular alcohol use; or an unstable medical condition (including uncontrolled diabetes mellitus or hypertension). If sleep apnoea symptoms were indicated via WatchPAT^™^ results, a report containing the results was sent to participants’ general practitioner and recommended further testing, but participants were allowed to remain in the study. Prior to any exercise, participants completed the Exercise and Sports Science Australia adult pre-exercise screening questionnaire. Prior to participation in any procedures, written informed consent was obtained. The study was approved by the Human Research Ethics Committee of Murdoch University, Australia (2020/207), and was retrospectively registered as a clinical trial with the Australian New Zealand Clinical Trials Registry (ACTRN12621001514897).

### Design and procedure

**Figure 1:**
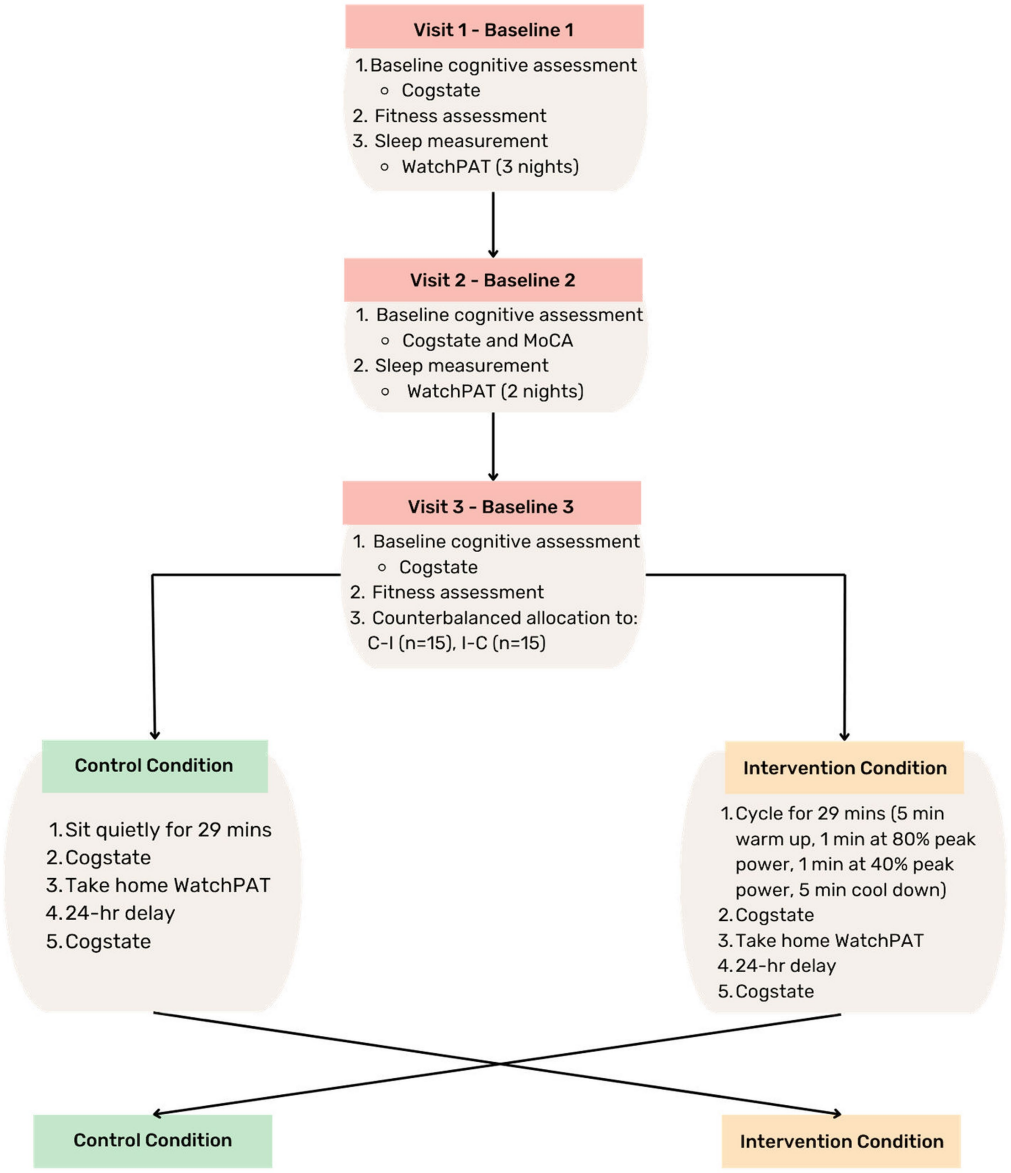
Study design. MoCA, Montreal Cognitive Assessment; C-I, control-intervention; I-C, intervention-control; min, minute; hr, hour.

A triple-baseline cognitive assessment, including Cogstate (described later) at all three occasions and MoCA only at baseline two, was completed followed by a two-period (AB, BA) crossover design, performed in a counterbalanced order (Figure 1). The triple baseline cognitive assessment was utilized to understand variability due to measurement error, based on previous repeatability recommendations of the Cogstate Brief Battery (Falleti et al., 2006), and to account for day-to-day variability in cognitive functioning. After cognitive assessments on visits one and three, participants underwent a fitness assessment on a cycle ergometer. Within the baseline period, sleep was measured for 5 nights, including at least one night of weekend sleep, using a take home device (WatchPAT^™^). Baseline visits were separated by at least 3 days to minimize practice and fatigue effects.

After baseline visits were complete, each participant underwent a single bout of acute high intensity cycling-based interval exercise, or a control condition where participants sat quietly for the same duration as the exercise session (29 minutes). Within 5 minutes of completing each condition, participants commenced cognitive assessment. Participants wore the WatchPAT^™^ device to measure sleep that night, and on the following morning participants returned to the laboratory to complete another cognitive assessment. Participants were asked to refrain from ingesting any substances that may impact sleep (the night before any session), and to maintain caffeine consumption within their usual habits the morning of all baseline and intervention sessions. For intervention sessions only, participants were also requested to refrain from any physical activity before the session.

### Baseline measures

#### Cardiorespiratory fitness measure

To measure cardiorespiratory fitness, participants completed a cycling-based test on an electromagnetically braked cycle ergometer (Velotron, Racermate, Seattle, Washington, United States) to measure peak aerobic capacity (VO_2peak_) and peak power output. A double baseline assessment of VO_2peak_ was conducted to gain the most accurate estimate of cardiorespiratory fitness (Tonino and Driscoll, 1988). The VO_2peak_ test protocol was based on Brown et al. (2017) and involved two-minute stages at incremental work rates no greater than two metabolic equivalents until exhaustion, based on the participants body mass. Participants >70 kg commenced at 30 W with increases of 20 W every stage. Participants between 70 and 100 kg commenced at 30 W with increases of 25 W every stage, and participants >100 kg commenced at 40 W with increases of 35 W every stage. The test was terminated at volitional exhaustion or when the cadence dropped below 60 revolutions per minute for 5 s. During the test, heart rate and ventilatory gasses were continuously recorded and averaged into 15-s intervals by a metabolic cart (TrueOne 2400, Parvomedics). VO_2peak_ was defined as the greatest 15-s mean value during the final 2 min of the test. Peak power output was calculated as the power at the last completed stage plus a pro-rata value of any uncompleted stage (Peiffer et al., 2008).

#### Self-report measures

Mood was measured using the Depression, Anxiety and Stress Scale (DASS; Lovibond and Lovibond, 1995). The PSQI was used to categorize individuals as ‘poor sleepers’ for inclusion, if their total score was >5, a threshold which has demonstrated high sensitivity and specificity (Backhaus et al., 2002). Years of education was determined via participant self-report and was a measure of full-time equivalent studies.

### Cognitive assessment

Cognitive domains of interest for the current study include executive function, attention and episodic memory, as these domains are most consistently associated with sleep and exercise (Nouchi et al., 2014; Gildner et al., 2019). Composite scores were created for each cognitive domain using tasks from Cogstate, a computerized cognitive assessment (see www.cogstate.com). Cogstate was selected as opposed to traditional pen-and-paper assessments with the aim of reducing learning and ceiling effects (e.g., words on the Cogstate shopping list change at each administration, compared to the California Verbal Learning test where words remain the same). The episodic memory composite was formed from the Continuous Paired Associate Learning task (number of errors; reverse scored), International Shopping List test recall, and One Card Learning test. The attention composite included the Detection and Identification tasks, and the executive function composite included the One Back and Groton Maze Learning tasks.

### Objective sleep assessment

A wrist-worn WatchPAT^™^ device (Itamar Medical, Caesarea, Israel) was used to measure baseline (5 nights, including at least one weekend night) and post-intervention sleep (one night). The WatchPAT^™^ device uses the peripheral arterial tone signal to detect sleep stages (Bresler et al., 2008). The WatchPAT^™^ has demonstrated sensitivity to detect light and deep sleep compared to the gold standard sleep measurement, polysomnography (80% agreement; Bresler et al., 2008). For the current study, measures of total sleep time (TST), sleep onset latency (in minutes), sleep efficiency (% of time in bed spent asleep), and % of REM sleep, light sleep (stages N1 and N2) and deep sleep (stage N3) were utilized. Scores were averaged over the five baseline nights to create a single baseline score for each sleep outcome of interest. If participants had missing baseline data, scores were averaged over the number of available nights of data (minimum 3 nights). Participants with more than two nights of missing sleep data were excluded from analyses which included sleep variables. A single night of sleep data was measured post-exercise and post-control. To account for night-to-night sleep variability, we included the within-participant standard deviation across baseline nights for each respective sleep variable as a covariate.

### Exercise intervention protocol

Participants completed a 5-min warm-up at 40% peak power output (determined via the average peak power of baseline fitness assessments) on the Velotron ergometer followed by ten 1-min intervals at 80% peak power output interspaced with 1-min intervals at 40% peak power output. After the intervals, participants completed a 4-min cooldown at 40% peak power output. The individualized power outputs were pre-programmed into, and controlled by, the ergometer’s built-in software.

### Control condition protocol

During the control condition, participants were asked to sit quietly in a lab room for the same amount of time as the exercise protocol (29 min). Participants were not permitted to use personal devices during this time and were instead provided with a set of magazines to read. On the day of the control condition, participants were asked to refrain from exercise for the whole day.

### Statistical analyses

**Figure 2:**
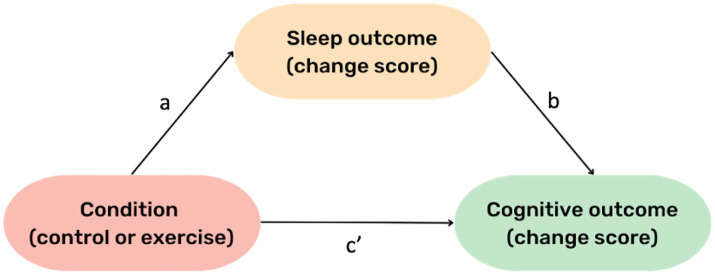
General Mediation Model. The “a” pathway represents the effect of condition (exercise vs. control) on change in the respective sleep outcome. The “b” pathway represents the effect of change in sleep on change in cognition, whilst holding condition constant (i.e., regardless of intervention condition). The c’ pathway represents the effect of intervention condition on cognition (~24 h later), while holding the respective sleep variable constant.

All analyses were conducted in R Version 4.2.1 (R Core Team, 2022). To address Aim one, a linear mixed model was conducted using condition (control, intervention) as the predictor variable, and cognitive change scores, from baseline to post-intervention, as the dependent variable (separate models for each cognitive domain), with participant identification number entered as a random factor. To address Aim two, another linear mixed model was conducted using condition (control, intervention) as the predictor variable, and sleep outcome change score, from baseline to post-intervention, as the dependent variable, with participant identification number entered as a random factor. Separate models were run for each sleep outcome, namely: TST, sleep onset latency, sleep efficiency, REM sleep %, light sleep %, and deep sleep %. To address Aim three, a mediation analysis was conducted, using the PROCESS macro for R (Hayes, 2017). Condition was treated as the independent categorical variable, sleep change score (baseline to post-intervention) as the mediator variable, and cognitive change score (baseline to 24 h post-intervention) as the dependent variable (Figure 2). Separate models were run for each sleep variable on each cognitive outcome. Age, sex, baseline fitness (averaged over the two assessments), and standard deviation of the baseline sleep measure per participant were treated as covariates in all models. Years of education and DASS score were tested as additional covariates, however their inclusion reduced model fit, and thus due to our limited statistical power, they were excluded from all analyses. For significant outcomes (determined by *p* < 0.05) from Aims one and two, the false discovery rate (FDR) correction was applied (Benjamini and Yekutieli, 2001). Aim three was the proof-of-concept aspect of our analysis, and therefore an FDR correction was not applied (Armstrong, 2014). Accordingly, for these results, we examined confidence intervals and magnitude of change as opposed to statistical significance. All results which showed a significant change in cognitive outcomes (after correction, if required) were compared to the smallest detectable change (SDC) score, detailed below.

Previous recommendations for sample size of a pilot trial are at least 9% of the sample size of the main planned trial (Cocks and Torgerson, 2013). Based on Fritz and Mackinnon (2007), a trial conducted using mediation analysis would require a sample of *n* = 116 (using bootstrapped corrected estimates and medium effect sizes), meaning the required sample for the current trial is minimum *n* = 10. Other studies have recommended pilot samples of at least 12 per group (Julious, 2005) and so we aimed to recruit *n* = 30 to maximize statistical power within each group and to account for potential attrition.

One participant had missing sleep data from baseline (only one viable night) and post-intervention nights due to failure of the sleep measurement device, these data were consequently removed from all analyses which included sleep variables (*n* = 29 for these analyses). This participant was included in analyses which did not use sleep data (i.e., analyses for Aim one) to maximize statistical power. There were an additional total 5 instances where the sleep measurement device failed on either the exercise or control night. These missing data were removed from all sleep analyses, however the remaining participant data were utilized (i.e., if the device failed only on the control night for one participant, sleep data from the night following exercise were retained). This missing data constituted 8.6% of the entire dataset, and three instances were after the control condition, while two instances were after the exercise condition.

#### Smallest detectable change

The triple baseline cognitive assessment was utilized to calculate change in cognition from baseline to post-exercise, not due to measurement error or expected fluctuation in cognitive performance; i.e., we have calculated the smallest detectable change (SDC; Bland and Altman, 1986). This methodology has been adopted from previous research (Geerinck et al., 2019). Firstly, the standard error of the mean (SEM) was calculated by creating a difference score between the second and third baseline cognitive measurements for each participant (second score – third score = difference). The difference between baseline two and three was selected based on the rationale that any differences between the first and second baseline will most likely be due to practice effects, but these effects will have plateaued by the third cognitive assessment (Falleti et al., 2006). Next, the standard deviation for the “difference” variable was calculated and divided by the square root of 2, resulting in the SEM ( $SEM=\frac{{SD}_{\mathrm{difference}}}{\sqrt{2}}$ ). Individual SDC (SDC_ind_) was then calculated using the following formula: SDC_ind_ = 1.96 * $\sqrt{2}$ * SEM. Finally, the group SDC (SDC_group_) was determined by dividing the SDC_ind_ by the square root of the number of subjects in the sample ( $\frac{{SCD}_{ind}}{\sqrt{n}}$ ), yielding a single SDC_group_ score for each cognitive composite. This SDC_group_ score was used to indicate whether the post-exercise changes in cognition are meaningful, or whether they may be due to measurement error. Namely, any exercise-induced significant changes at the group level were compared to the SDC_group_ score. Further, because there is significant intra-individual variability in cognitive response to exercise, the SDC_ind_ for the respective cognitive variable was ± to/from each individual participant’s “best score” across the baseline cognitive measurement. This methodology resulted in an individual threshold which each participant would need to reach to achieve probable meaningful cognitive change.

## Results

Baseline participant demographics are presented in Table 1. Briefly, the cohort had a mean age of 69.2 ± 4.3 years, was comprised of 66% females, and as per inclusion criteria, all participants were poor sleepers (>5 on the PSQI).

**Table 1:** Participant characteristics (*n* = 30).

Demographics	Baseline
Age, mean (SD)	69.2 (4.3)
Sex, % female (*n*)	66.7 (20)
Years of education, mean (SD)	14 (2.7)
Mood (DASS score), mean (SD)	9.1 (4.7)
MoCA score, mean (SD)	26.4 (1.8)
Cardiorespiratory fitness (VO_2peak_)	23 (5.6)
Sleep medication use, % positive (*n*)	50 (15)
Total PSQI score, mean (SD), range	9.7 (2.6), 6–15
Sleep efficiency (%), mean (SD)	84.9 (5.3)
Total sleep time (hours), mean (SD)	6.5 (0.8)
Sleep onset latency (minutes), mean (SD)	19.5 (5.6)
Light sleep (%), mean (SD)	63.2 (8.6)
Deep sleep (%), mean (SD)	14.3 (4.3)
REM sleep (%), mean (SD)	22.5 (5.7)

DASS, Depression, Anxiety and Stress Scale; PSQI, Pittsburgh Sleep Quality Index; MoCA, Montreal Cognitive Assessment; REM, rapid eye movement. All sleep variables taken from baseline WatchPAT measurements, except for PSQI score which is self-reported. VO_2peak_ refers to peak aerobic capacity, averaged across two baseline fitness tests.

### Effect of the exercise intervention on cognitive function and sleep

Linear mixed models showed no between condition differences (intervention vs. control) for change in any cognitive outcome from pre- to immediately post-intervention (Table 2). Similarly, there were no differences in changes in sleep variables between control and intervention conditions (Table 2).

**Table 2:** Linear mixed model analyses for the effect of condition (control vs. intervention) on sleep and cognitive outcomes (analyzed separately for each outcome).

Respective outcome	β	SE	*p*-value
Episodic memory	−0.05	0.18	0.776
Attention	−0.02	0.19	0.926
Executive function	−0.03	0.14	0.836
Total sleep time (hours)	0.10	0.20	0.617
Number of awakenings	1.43	0.83	0.091
Sleep onset latency (mins)	−3.47	2.73	0.210
Sleep efficiency (%)	0.39	0.95	0.686
REM sleep (%)	−0.44	1.50	0.772
Light sleep (%)	1.53	1.81	0.402
Deep sleep (%)	−0.88	0.95	0.361

β, Unstandardized Regression Coefficient; SE, Standard Error; REM, Rapid Eye Movement. Control condition used as reference group. Age, sex, baseline fitness and standard deviation of the respective sleep variable used as covariates in all models.

### Sleep as a mediator for associations between exercise intervention and cognition

Mediation analyses demonstrated no indirect effects of intervention condition on change in any cognitive domain through change in any sleep variable (Table 3). However, regardless of intervention condition, a small effect was observed where increases in deep sleep % from baseline predicted a positive change in episodic memory performance the following day (“b” pathway, Figure 2; β = 0.03, SE = 0.03, [95% CI 0.001–0.066]). Conversely, small increase in light sleep % from baseline predicted a small negative change in episodic memory performance the following day (“b” pathway, Figure 2; β = −0.02, SE = 0.01, [95% CI −0.04 to −0.004]). However, because this was a proof-of-concept aim, conclusions regarding statistical significance cannot be drawn.

**Table 3:** Indirect effects of the intervention condition (control vs. exercise) on cognitive outcomes (change scores) through sleep variables (change scores).

	Executive function		Episodic memory		Attention	
	Effect	95% CI	Effect	95% CI	Effect	95% CI
Total sleep time (hours)	0.01	−0.05, 0.08	0.002	−0.06, 0.05	0.01	−0.07, 0.16
Number of awakenings	0.003	−0.13, 0.10	−0.03	−0.13, 0.05	−0.06	−0.23, 0.07
Sleep onset latency (mins)	0.04	−0.03, 0.14	0.05	−0.04, 0.19	0.06	−0.04, 0.25
Sleep efficiency (%)	0.001	−0.07, 0.06	0.002	−0.06, 0.04	0.02	−0.12, 0.17
REM sleep (%)	0.001	−0.04, 0.06	−0.01	−0.12, 0.07	−0.01	−0.14, 0.12
Light sleep (%)	0.02	−0.05, 0.11	−0.04	−0.18, 0.07	−0.03	−0.16, 0.12
Deep sleep (%)	0.03	−0.06, 0.14	−0.03	−0.17, 0.05	−0.04	−0.17, 0.08

CI, Confidence Interval; REM, Rapid Eye Movement. Control condition used as reference group. All models adjusted for age, sex, baseline fitness and baseline standard deviation of the respective sleep variable. Change scores refer to change from baseline to 24 h post intervention for cognition, and baseline to the night following intervention for sleep.

### Smallest detectable change outcomes

**Figure 3:**
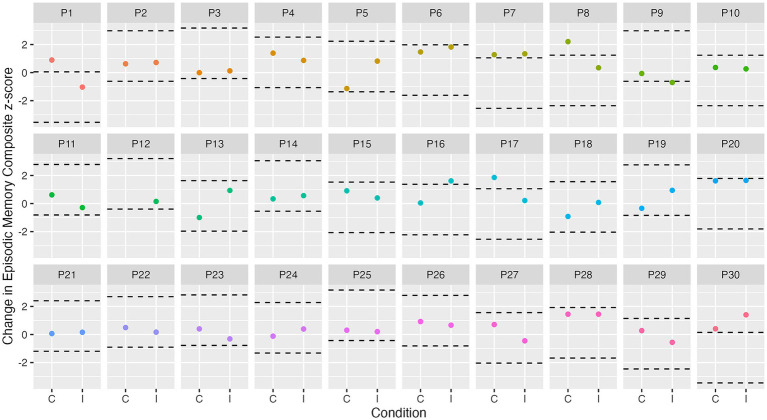
Individual change in episodic memory from baseline to immediately post-intervention. Dashed lines represent ± SDC_ind_ (z-score of 1.8) for episodic memory from each participant’s best baseline episodic memory score. C, control condition; I, intervention condition; P, participant number.

**Figure 4:**
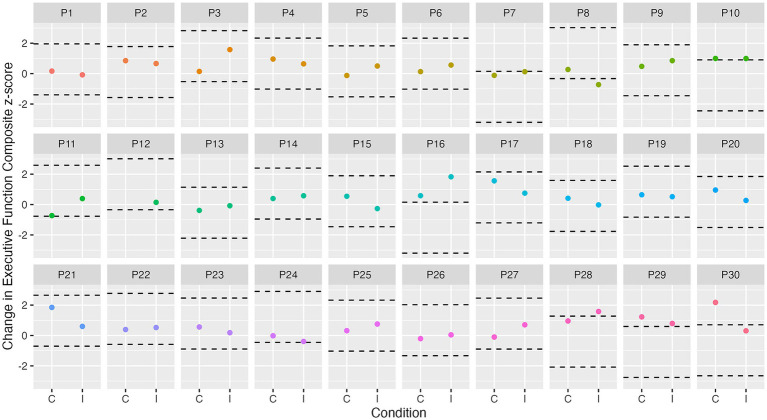
Individual change in executive function from baseline to immediately post-intervention. Dashed lines represent ± SDC_ind_ (z-score of 1.7) for executive function from each participant’s best baseline executive function score. C, control condition; I, intervention condition; P, participant number.

**Figure 5:**
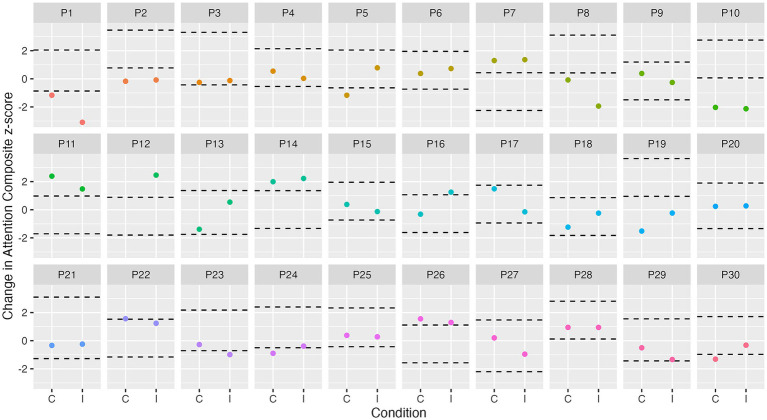
Individual change in attention from baseline to immediately post-intervention. Dashed lines represent ± SDC_ind_ (z-score of 1.3) for attention from each participant’s best baseline attention score. C, control condition; I, intervention condition; P, participant number.

At the group level, a cognitive composite score change of 0.24 for attention, 0.30 for executive function, and 0.32 for episodic memory is required to observe “true change” (SDC_group_). At the individual level, a change of 1.34 for attention, 1.67 for executive function, and 1.80 for episodic memory is required to observe “true change” (SDC_ind_). Figures 3–5 show individual participant change in episodic memory, executive function and attention cognitive composite scores, respectively, from baseline to immediately post-intervention, compared to their individual meaningful change threshold, calculated using the SDC_ind_. Across a total of 60 assessments (one post-exercise, one post-control for each participant), participants showed meaningful change from baseline to immediately post-intervention for both episodic memory and executive function 15% of the time, and for attention 42% of the time. Table 4 details these frequencies by intervention condition and categorized as “positive” or “negative” score change. Supplementary Figures S1–S3 and Supplementary Table S1 detail these same analyses for cognitive score change from baseline to 24-h post-intervention.

**Table 4:** Frequency table of meaningful cognitive change from baseline to immediately post-intervention.

	Episodic memory		Executive function		Attention	
	Control	Exercise	Control	Exercise	Control	Exercise
Increase	5	3	4	4	5	6
Decrease	0	1	0	1	8	6
No change	25	26	26	25	17	18

“Increase” means participants increased performance from baseline to a greater extent than the smallest detectable change. “Decrease” means participants decreased performance from baseline to a greater extent than the smallest detectable change. “No change” means participants change score fell within the range of the smallest detectable change.

## Discussion

The current study was intended as an exploratory, proof-of-concept trial to examine associations between acute exercise, objectively measured sleep, and cognitive function in cognitively unimpaired older adults. High intensity exercise did not affect any sleep variable, or cognitive function immediately post-exercise. For our proof-of-concept aim, we observed very little evidence for an indirect (mediating) effect of any sleep variable on associations between exercise and cognition (indicated by small effects and wide confidence intervals in Table 3). We did observe that regardless of acute exercise, small changes in light sleep % and deep sleep % from baseline to post-intervention were associated with changes in episodic memory from baseline to 24-h post-intervention, such that increased light sleep and decreased deep sleep were associated with poorer episodic memory.

We observed no influence of our acute exercise intervention on immediate cognitive performance, which is surprising given exercise can have immediate physiological and cognitive impacts (Coelho et al., 2014; Chang et al., 2019; McSween et al., 2019). Our results may be partially explained by the use of a triple baseline assessment of cognitive function. This methodology aimed to better account for variability in day-to-day cognitive functioning, as opposed to a single baseline measure (Bielak et al., 2017). As a result, any exercise-induced change in cognition may not have been large enough to differ significantly from natural intra-individual variability. Indeed, our individual analyses (see Figures 3–5) support this notion, showing that only a small number of individuals showed any meaningful change from baseline. Additionally, most previous studies conduct baseline cognitive assessments on the day of the intervention (e.g., Chang et al., 2014), which was not feasible in the current study due to the triple baseline measure (which could not be conducted on a single day, given that we wanted to utilize data from all three assessments in the baseline score). Finally, although participants in the current study were asked to maintain consistent caffeine and morning routine habits on the day of the session, there may be some variability (e.g., in social interactions) which could have influenced results (Bielak et al., 2017).

Contrary to our hypothesis, our results also showed no effect of exercise on any sleep variable, compared to baseline. These results are again unexpected, as previous research shows beneficial effects of acute exercise on multiple sleep parameters (Kredlow et al., 2015; Larsen et al., 2019). However, it should be noted that the effect sizes reported in Kredlow et al. were relatively small (Cohen’s *d* = 0.17–0.25), thus the current study may have been underpowered to detect such changes (powered for *d* = 0.60). Additionally, we examined sleep change from baseline to post-intervention and compared to control, whereas previous studies have examined acute exercise vs. non-exercise days, without characterizing baseline sleep of the sample (Kredlow et al., 2015). Baseline comparisons are important in this context because of night-to-night sleep variability, which may be especially prevalent in older adults with sleep complaints (Kay et al., 2013). Although we did account for night-to-night sleep variability in our statistical analyses, large variability within some participants may still influence overall findings, particularly within our small sample. Finally, the current study recruited individuals who were self-reported “poor” sleepers, however data from our objective measures showed sleep characteristics falling within the normal range (e.g., mean 6.5 h TST; mean 84.9% sleep efficiency). Indeed, previous research indicates that subjective and objective sleep assessments may measure, equally important, but different aspects of sleep; both are associated with health outcomes (Lou et al., 2015; Wirth et al., 2015; Sewell et al., 2021). Thus, the discrepancy between objective and subjective sleep in the current research may partially explain our null results, as our exercise intervention may not have been sufficient to induce detectable changes in sleep. Further research is needed to examine the effects of both acute and chronic exercise on objectively measured sleep in a larger sample of older adults, using both objective and self-report sleep measures, considering night-to-night sleep variability.

In contrast to previous research (Wilckens et al., 2018; Cheval et al., 2022), we observed very little change in sleep and cognition from our exercise intervention, indicating there was not a mediating (indirect) effect. These results may be due to the lack of effect of exercise on sleep (“a” pathway, Figure 2), as we did observe associations between sleep and cognition (“b” pathway, Figure 2). Potential explanations of the null effect of exercise on sleep in our cohort are detailed above. However, it is important to consider that this is the first experimental study to test a mediating effect of sleep on associations between exercise and cognition, and was intended as a proof-of-concept trial, thus we cannot draw conclusions regarding statistical significance. From our results we see that the magnitude of our indirect effects was very small (Table 3). One previous observational study also reported null indirect effects for the impact of physical activity on cognition through sleep (Yuan et al., 2020). Thus, it is crucial to further test this mediation model using objectively measured sleep in an experimental design to determine cause-effect associations between these variables. Such studies should employ larger sample sizes because the effect size of exercise on sleep is already relatively small (Kredlow et al., 2015), and combined with the requirement of large samples to detect a mediating effect, power is an important consideration for such trials (Fritz and Mackinnon, 2007). Such power would also allow for statistical consideration of sleep variability, omission of which may contribute to a null effect of exercise on sleep.

From our mediation analysis, we observed that, regardless of exercise, sleep may be related to cognition. Specifically, small increases in deep sleep % (stage N3) from baseline to post-intervention were associated with small improvements in episodic memory, whereas increased light sleep % (stages N1 and N2) was associated with poorer episodic memory performance. Again, because this was a proof-of-concept trial, we cannot make decisions regarding statistical significance. However, these results are consistent with the broader literature which shows that habitual sleep is associated with cognitive function (Scullin and Bliwise, 2015; Dzierzewski et al., 2018). More specifically, a recent meta-analysis showed that memory and executive function are the two cognitive domains most frequently associated with sleep parameters, in both single-night and multi-night studies (Qin et al., 2023). However, in contrast to the current results, this meta-analysis did not examine associations between light sleep % and specific cognitive domains (due to a lack of studies investigating these relationships). Importantly, many studies in this area have not examined change in sleep from a baseline characterization, which is important because of night-to-night sleep variability. Thus, our study builds on the current literature by showing that changes in specific sleep stages may be associated with change in particular cognitive domains, however further research which is adequately powered to test such hypotheses is required. Despite associations between deep sleep and memory in older adults being inconsistent in previous studies (Qin et al., 2023), the current study supports the overall notion that age-related decreases in deep sleep % and increases in light sleep % are associated with negative changes in memory. Further research is required to characterize longitudinal associations between age-related changes in sleep and cognitive function.

In the current study, we utilized a triple baseline cognitive assessment with the aim of overcoming measurement error and accounting for natural variability in cognitive performance. Our group level analyses showed no exercise-induced cognitive change, and examining individual level data showed a small number of individual performance increase (see Figures 3–5; Table 4). However, our results do provide an indication of the smallest meaningful change needed for future studies using Cogstate to assess cognition in similar cognitively unimpaired older adult populations. The SDC_group_ and SDC_ind_ scores determined in this study for each cognitive composite may be utilized in future research to determine if post-intervention change is meaningful. For example, our SDC_group_ results showed that a change of 0.32 z-score units is required on our episodic memory composite score to be confident that such change is not due to measurement error. Based on our results from the mediation analysis, an approximate 10% change in deep sleep from baseline to post-intervention would be required to produce a 0.32-unit change in the episodic memory composite score. Similarly, an approximate 16% change in light sleep would be required to produce this change in episodic memory. Given the average variability across baseline sleep nights was 2.98% for deep sleep, and 5.86% for light sleep, the required change is quite large, and would be unlikely to occur acutely in the current sample. These results support the notion that the effects of sleep on cognition may be relatively small in an acute setting, in a population of cognitively unimpaired older adults who report poor sleep, and highlight the need for future studies to better quantify observed cognitive change, and consider whether such change is meaningful.

We acknowledge that this proof-of-concept study is subject to limitations, including small sample size, resulting in limited power to detect a mediating effect of sleep on the relationship between exercise and cognition. Given that there is cross-sectional evidence for a mediating effect of sleep on the relationship between exercise and cognition, future studies should consider testing this model in a larger sample, with a chronic exercise intervention. Further, the use of a chronic exercise intervention would allow additional nights of sleep measurement post-intervention, as opposed to the single night measure in the current study. Our sample was also relatively homogenous, comprising highly motivated, highly educated, and physically active individuals. However, we did utilize a well-validated objective sleep measure with the ability to examine sleep staging, a comprehensive cognitive assessment with a triple baseline, and a fully supervised exercise intervention. Moreover, while we were not able to control whether participants exercised on days where baseline sleep was measured, we did account for this using a physical activity diary.

The current study showed that an acute high intensity exercise intervention did not yield immediate post-intervention impacts on cognition or sleep in cognitively unimpaired older adults with self-reported poor sleep. However, sleep appears to be associated with cognition independently of acute exercise; an association which may be dependent upon the specific sleep and cognitive variables being examined. Further research is required to elucidate associations between exercise, sleep and cognition in older adults, namely examining a mediating effect of sleep in chronic exercise intervention studies. Such research should also consider potential biological explanations for these associations, e.g., levels of brain-derived neurotropic factor (BDNF) which are influenced by both sleep and exercise. This type of research will contribute to knowledge of the most effective interventions for preserving cognitive function in our aging population.

## Data availability statement

The raw data supporting the conclusions of this article will be made available upon reasonable request by the authors, without undue reservation.

## Ethics statement

The studies involving humans were approved by Murdoch University Human Research Ethics Committee. The studies were conducted in accordance with the local legislation and institutional requirements. The participants provided their written informed consent to participate in this study.

## Author contributions

KS contributed to data collection, conception of the project, data analysis, drafting, and revising manuscript. NS contributed to project design and data collection, and wrote sections of the manuscript. SR-S, HS, JP, and KE contributed to conception and design of the project and data interpretation. BB contributed to supervision, study design, data interpretation, and wrote sections of the manuscript. All authors contributed to the article and approved the submitted version.
