# Supplementary material for: The effect of acute exercise on objectively measured sleep and cognition in older adults
Source: Front Psychol. 2023 Oct 5;14:1207199. doi: 10.3389/fpsyg.2023.1207199 (PMC10585032; doi:10.3389/fpsyg.2023.1207199)
Supplement: Supplementary file 1 [file Data_Sheet_1.docx]

#
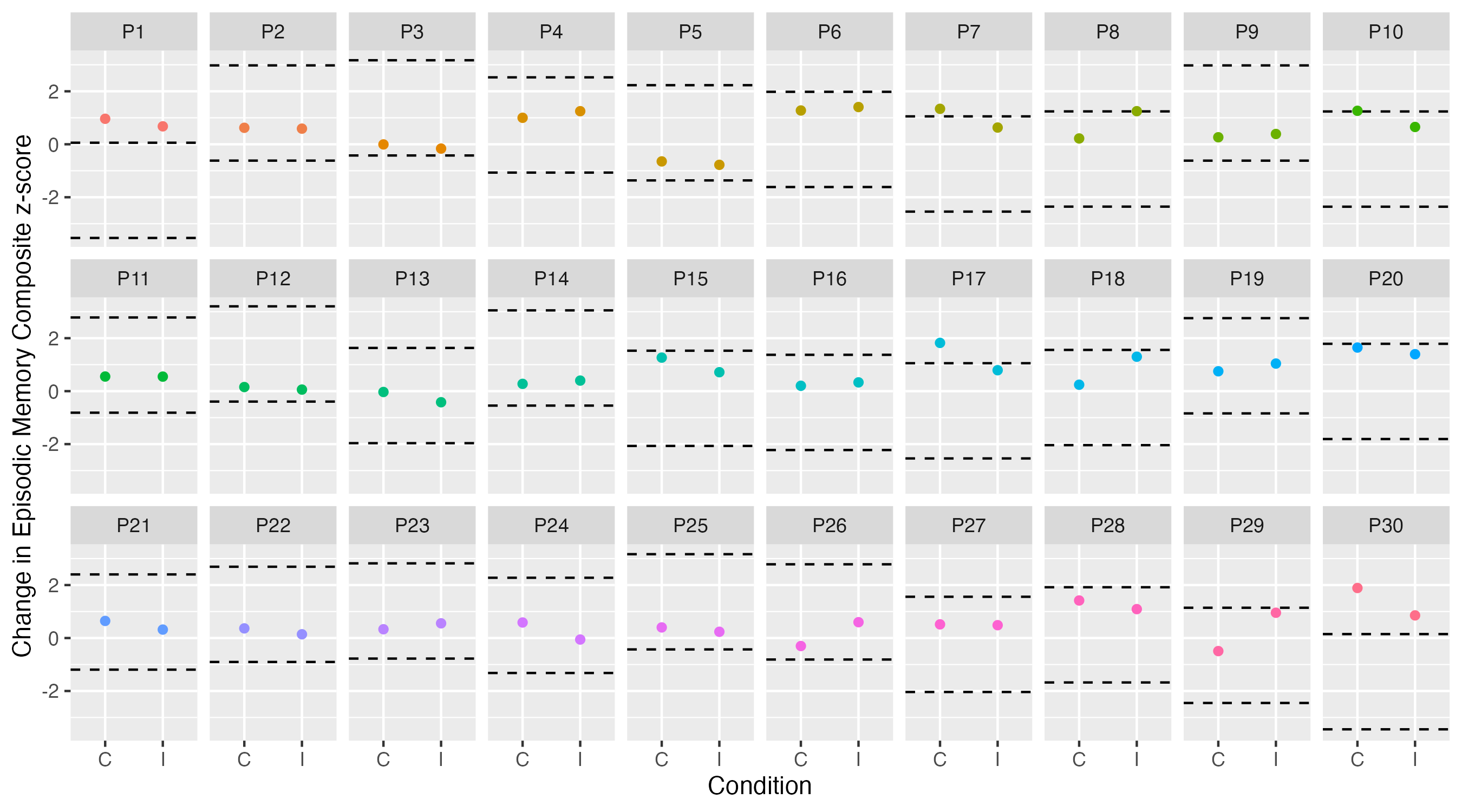
**SUPPLEMENTARY MATERIALS**

Supplementary Figure 1. Individual change in episodic memory from baseline to 24-hours post-intervention. Dashed lines represent +/- SDC_ind_ (z-score of 1.8) for episodic memory from each participant’s best baseline episodic memory score. Abbreviations: C = control condition, I = intervention condition, P = participant number.


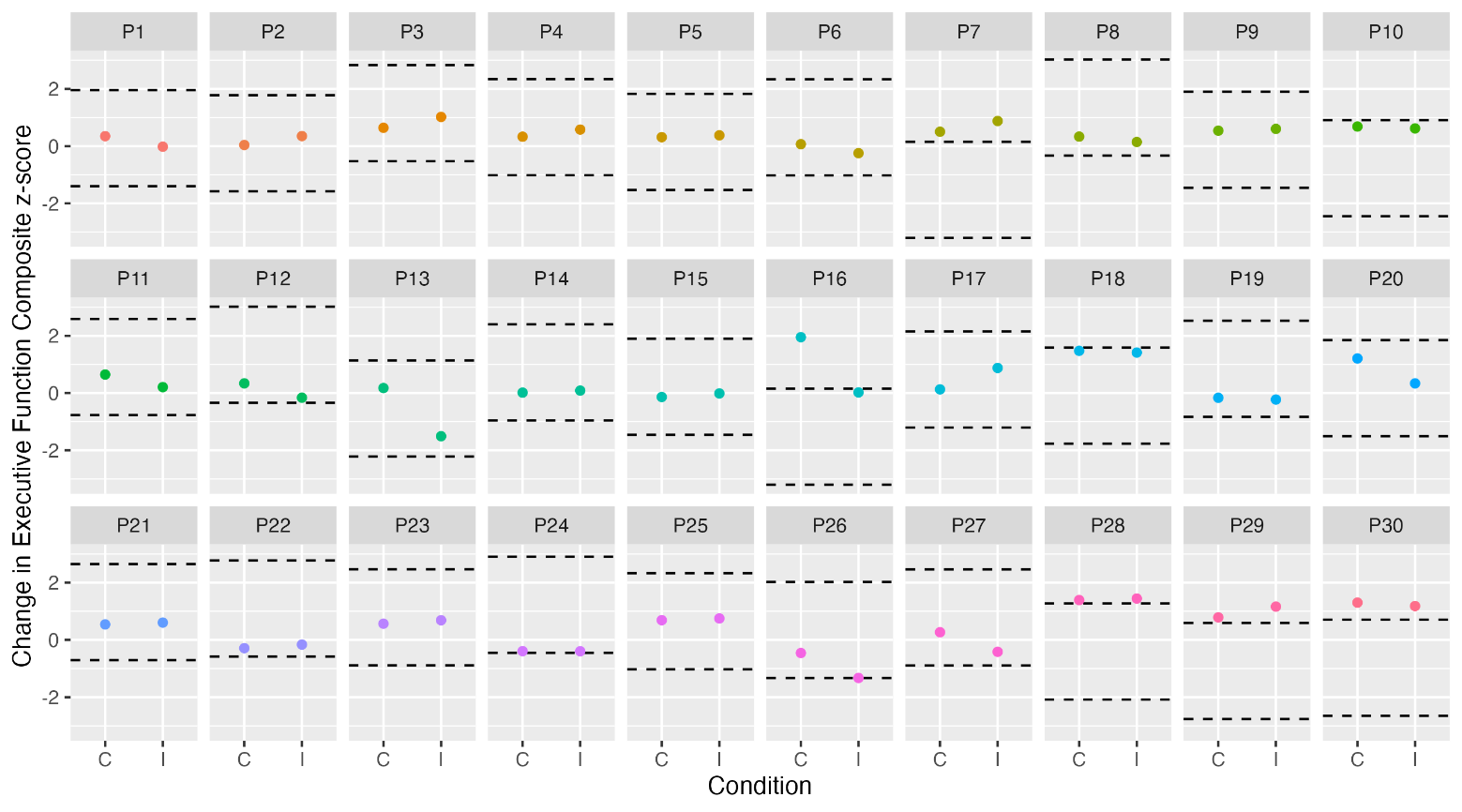


Supplementary Figure 2. Individual change in executive function from baseline to 24-hours post-intervention. Dashed lines represent +/- SDC_ind_ (z-score of 1.7) for executive function from each participant’s best baseline executive function score. Abbreviations: C = control condition, I = intervention condition, P = participant number.


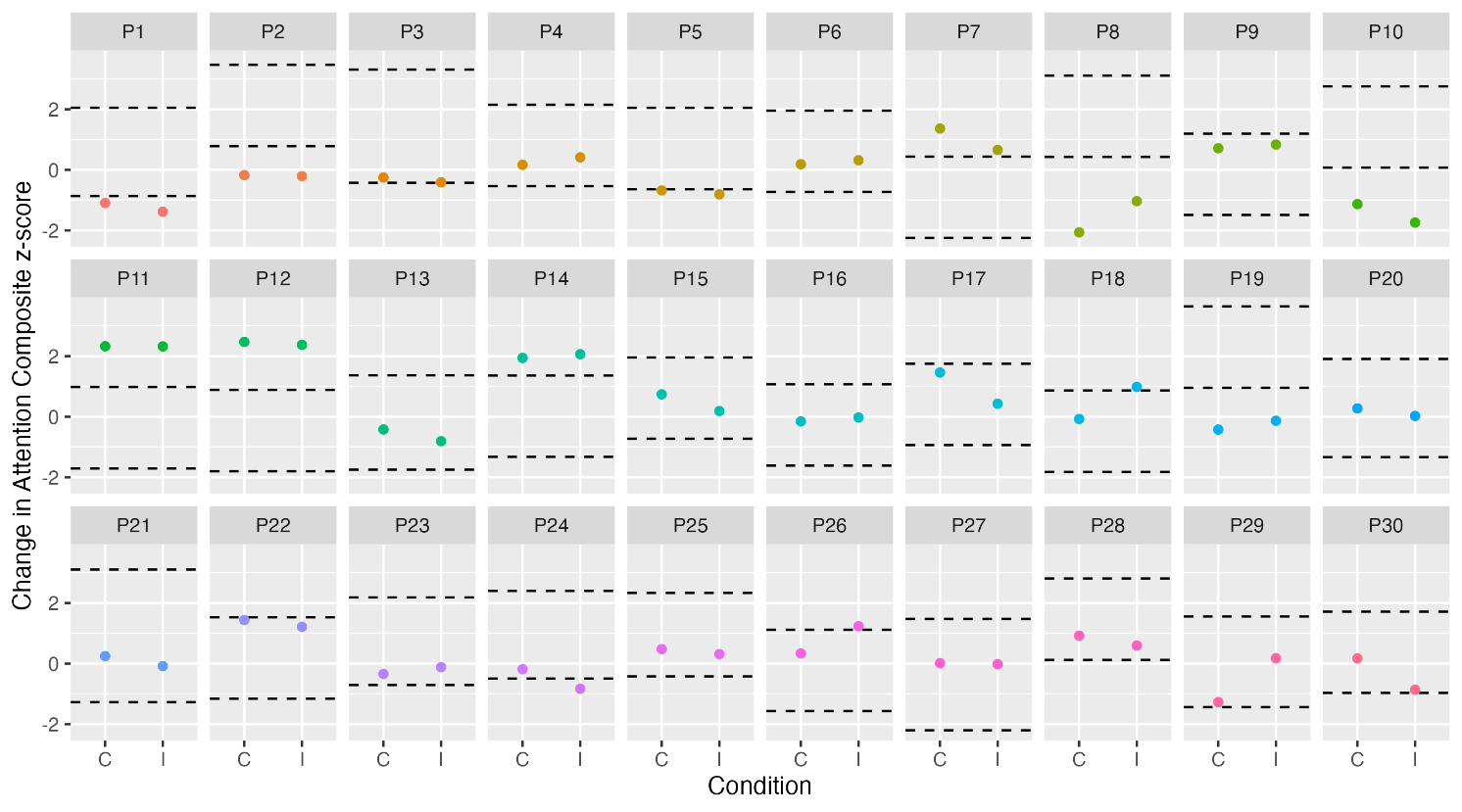


Supplementary Figure 3. Individual change in attention from baseline to 24-hours post-intervention. Dashed lines represent +/- SDC_ind_ (z-score of 1.3) for attention from each participant’s best baseline attention score. Abbreviations: C = control condition, I = intervention condition, P = participant number.

**Supplementary Table 1.** Frequency table of meaningful cognitive change from baseline to 24-hours post-intervention.

|  | Episodic Memory | |  | Executive Function | |  | Attention | |
| --- | --- | --- | --- | --- | --- | --- | --- | --- |
|  | Control | Exercise |  | Control | Exercise |  | Control | Exercise |
| Increase | 5 | 3 |  | 5 | 4 |  | 4 | 6 |
| Decrease | 0 | 0 |  | 0 | 0 |  | 6 | 7 |
| No Change | 25 | 27 |  | 25 | 26 |  | 20 | 17 |

‘Increase’ means participants increased performance from baseline to a greater extent than the smallest detectable change. ‘Decrease’ means participants decreased performance from baseline to a greater extent than the smallest detectable change. ‘No change’ means participants change score fell within the range of the smallest detectable change.
